# Cellular Mechanisms of Alpha Herpesvirus Egress: Live Cell Fluorescence Microscopy of Pseudorabies Virus Exocytosis

**DOI:** 10.1371/journal.ppat.1004535

**Published:** 2014-12-04

**Authors:** Ian B. Hogue, Jens B. Bosse, Jiun-Ruey Hu, Stephan Y. Thiberge, Lynn W. Enquist

**Affiliations:** 1 Department of Molecular Biology, Princeton University, Princeton, New Jersey, United States of America; 2 Princeton Neuroscience Institute, Princeton University, Princeton, New Jersey, United States of America; Northwestern University, United States of America

## Abstract

Egress of newly assembled herpesvirus particles from infected cells is a highly dynamic process involving the host secretory pathway working in concert with viral components. To elucidate the location, dynamics, and molecular mechanisms of alpha herpesvirus egress, we developed a live-cell fluorescence microscopy method to visualize the final transport and exocytosis of pseudorabies virus (PRV) particles in non-polarized epithelial cells. This method is based on total internal reflection fluorescence (TIRF) microscopy to selectively image fluorescent virus particles near the plasma membrane, and takes advantage of a virus-encoded pH-sensitive probe to visualize the precise moment and location of particle exocytosis. We performed single-particle tracking and mean squared displacement analysis to characterize particle motion, and imaged a panel of cellular proteins to identify those spatially and dynamically associated with viral exocytosis. Based on our data, individual virus particles travel to the plasma membrane inside small, acidified secretory vesicles. Rab GTPases, Rab6a, Rab8a, and Rab11a, key regulators of the plasma membrane-directed secretory pathway, are present on the virus secretory vesicle. These vesicles undergo fast, directional transport directly to the site of exocytosis, which is most frequently near patches of LL5β, part of a complex that anchors microtubules to the plasma membrane. Vesicles are tightly docked at the site of exocytosis for several seconds, and membrane fusion occurs, displacing the virion a small distance across the plasma membrane. After exocytosis, particles remain tightly confined on the outer cell surface. Based on recent reports in the cell biological and alpha herpesvirus literature, combined with our spatial and dynamic data on viral egress, we propose an integrated model that links together the intracellular transport pathways and exocytosis mechanisms that mediate alpha herpesvirus egress.

## Author Summary

Pseudorabies virus, an alpha herpesvirus, is an important veterinary pathogen, and related to human varicella-zoster virus and herpes simplex viruses. New alpha herpesvirus particles are assembled inside an infected cell, and must exit from the infected cell by taking advantage of cellular mechanisms. How these virus particles are transported inside the infected cell and secreted at the cell surface is not understood in great detail. In particular, how this process unfolds over time is not easily observed using previous methods. In this study, we developed a new method to observe this egress process. Using this method, we described how virus particles move on their way out: individual virus particles travel to the cell surface, directly to the exit site, where they pause for several seconds before crossing out of the cell. We identified several cellular proteins that are involved in this process. After exiting, virus particles remained stuck to the outer cell surface. Finally, we draw connections between our observations and other recent studies to propose an integrated model of how alpha herpesvirus particles exit from infected cells.

## Introduction

Pseudorabies virus (PRV; suid herpesvirus 1) is a veterinary pathogen, widely used as a neuroanatomical tracing tool, and related to the human alpha herpesviruses varicella-zoster virus (VZV) and herpes simplex virus 1 and 2 (HSV-1 & -2). Transport and egress of newly assembled alpha herpesvirus particles is a highly dynamic process involving viral components working in concert with host membrane transport systems. After capsid assembly and genome packaging in the nucleus, particles exit the nucleus by budding through the inner and outer nuclear membranes (reviewed in [1]). Viral membrane proteins are produced in the secretory pathway and traffic to the site of secondary envelopment, thought to be trans-Golgi [2]–[4] and/or endosomal membranes [5], [6]. Virus particles acquire their envelopes by budding into these membranes, producing an enveloped virion inside an intracellular vesicle. This virion transport vesicle then traffics to the plasma membrane, where the virion exits the infected cell by exocytosis. While this general description of viral egress is widely accepted, the specific mechanisms involved are not well studied.

To elucidate the location, dynamics, and molecular mechanisms of alpha herpesvirus egress, we developed a live-cell fluorescence microscopy method to visualize the final steps in PRV particle transport and exocytosis. This method takes advantage of total internal reflection fluorescence (TIRF) microscopy to selectively image particle dynamics near the plasma membrane, and a pH sensitive fluorescent probe that reveals the precise moment and location of exocytosis.

We characterized particle movement by single-particle tracking and mean squared displacement (MSD) analysis. We found that particles are tightly confined at the plasma membrane before and after exocytosis, and undergo a sharp movement during the tens of milliseconds immediately after pHluorin dequenching.

Previous studies in the HSV-1 literature sought to identify Rab proteins involved in alpha herpesvirus replication. Rab GTPases regulate essentially all intracellular membrane traffic. In their active GTP-bound form, Rab proteins bind to intracellular membranes, recruit molecular motors, and interact with a wide variety of effector proteins that mediate docking and fusion with target membranes [7]. The Elliott and Lippé laboratories screened siRNAs targeting Rab proteins [5], [6], [8], and Zenner et al. screened a panel of putative Rab GTPase activating proteins (RabGAPs) [9]. Collectively, these studies showed that many different Rab proteins – ER, Golgi, endocytic, and secretory Rabs – are involved in virus replication. However, a fundamental limitation of such loss-of-function screens is that it remains unclear whether these Rab proteins are involved directly in viral egress, or have other indirect or upstream effects. To resolve this ambiguity, we imaged Rab proteins in live cells to identify only those that are spatially and dynamically associated with viral exocytosis.

We also investigated the plasma membrane sites of viral exocytosis, and found no evidence for specialized cytoskeleton-depleted exocytosis sites, contrary to a previous report on HSV-1 [10]. However, we did observe that viral exocytosis events occur most frequently near plasma membrane patches of LL5β, part of a complex that anchors microtubules to the plasma membrane and regulates exocytosis. Finally, based on previous reports in the cell biological and alpha herpesvirus literature combined with our present findings, we propose an integrated model that links together the intracellular transport pathways and exocytosis mechanisms that mediate alpha herpesvirus egress.

## Results

### Validation of Fluorescent Constructs

To visualize virus particle exocytosis, we genetically fused superecliptic pHluorin [11], a pH-sensitive green fluorescent protein (GFP), into the predicted first extracellular loop of PRV glycoprotein M (gM) to create gM-pHluorin. The wild-type gM locus was replaced with the gM-pHluorin coding sequence in wild-type PRV Becker, and PRV 180, which expresses a red fluorescent small capsid protein VP26 (mRFP capsid [12]), creating PRV 486 and PRV 483, respectively.

**Figure 1:**
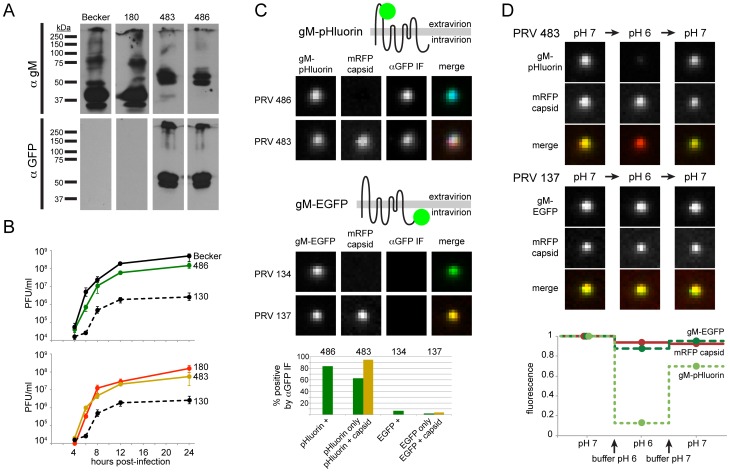
Characterization of PRV recombinants. (A) Cells infected with parental viruses (PRV Becker, 180) or gM-pHluorin expressing viruses (PRV 483, 486) were harvested at 12 hpi, and lysates were analyzed by Western blot. Parallel blots were probed with polyclonal antiserum against gM (αgM), or a monoclonal antibody that recognizes pHluorin (αGFP). (B) Single-Step Virus Replication. Parallel cell cultures were infected in triplicate with the indicated parental viruses (PRV Becker, 180), gM-pHluorin expressing viruses (PRV 483, 486), or a gM-null virus (PRV 130). Cells and supernatants were harvested at indicated times, and infectious virus titer was measured by plaque assay. Error bars represent range. (C) Membrane Topology. Particles produced by the indicated viruses were imaged to detect gM-pHluorin or gM-EGFP, mRFP capsid, and immunofluorescence targeting the pHluorin or EGFP epitopes (αGFP IF). Immunofluorescence labeling was performed without membrane permeabilization. The schematic represents the predicted topology of gM-pHluorin or gM-EGFP. Images depict single representative virus particles (each image is 2.5 µm by 2.5 µm). Bar graph represents classification and quantification of particles based on fluorescence (n≥237 particles per condition). (D) pH Sensitivity. Particles produced by the indicated viruses were imaged to detect gM-pHluorin or gM-EGFP, and mRFP capsid after addition of buffers at pH 6 or 7. Images depict single representative virus particles (each image is 2.5 µm by 2.5 µm). Graph represents relative particle fluorescence after each indicated buffer change (n≥154 particles per condition).

To validate gM-pHluorin expression, we probed parallel Western blots with anti-gM and anti-GFP antibodies. The major gM-pHluorin bands were detected by both antibodies, and were shifted by about 30 kDa, consistent with fluorescent protein fusion (Figure 1A). In single-step replication, PRV 486 and PRV 483 exhibited modest defects relative to parental viruses, but replicated much better than PRV 130, a gM-null strain (Figure 1B).

### gM-pHluorin Membrane Topology

gM is predicted to be an 8-pass transmembrane protein, oriented with its N- and C-termini on the cytosolic/intravirion side of membranes ([13], see schematic in Figure 1C). To confirm the membrane topology of gM-pHluorin, we performed immunofluorescence (IF) labeling of freshly-prepared infected cell supernatants, using an anti-GFP antibody without membrane permeabilization (Figure 1C). In PRV 483 supernatants, 95% of particles containing both gM-pHluorin and mRFP capsids were labeled by IF, indicating that the pHluorin epitope is exposed on the outside of the virion envelope. As a control, PRV 134 and PRV 137 express gM with a C-terminal EGFP fusion (gM-EGFP). Fewer than 6% of particles containing gM-EGFP were labeled by IF, indicating that the EGFP moiety is not exposed on the virion surface (Figure 1C). Together, these results confirm the predicted membrane topology of our gM fluorescent protein fusions.

We also observed many gM-pHluorin puncta that do not contain capsids. These may represent previously described “light” or “L-particles” [14], [15], which are expected to have the same membrane topology as a virion. These puncta without capsids may also consist of cellular debris, likely with variable membrane topology. In PRV 483 supernatants, 63% of puncta without capsids were labeled by IF, suggesting that this population is a heterogeneous mix of L-particles and cellular debris.

### gM-pHluorin Is a Sensitive Probe of Extravirion pH

To validate the pH sensitivity of gM-pHluorin, we imaged virus particles after adding saline buffers of pH 6 or pH 7. Particles exhibited strong but reversible changes in pHluorin fluorescence dependent on pH, but mRFP capsid fluorescence was mostly unaffected (Figure 1D). As a control, particles containing gM-EGFP exhibited little change in green fluorescence, as EGFP is inherently less pH sensitive, and may be somewhat protected from pH changes by the virion envelope (Figure 1D). These results show that gM-pHluorin is a highly sensitive probe of extravirion pH.

### Live-Cell Fluorescence Microscopy of Particle Transport and Egress

**Figure 2:**
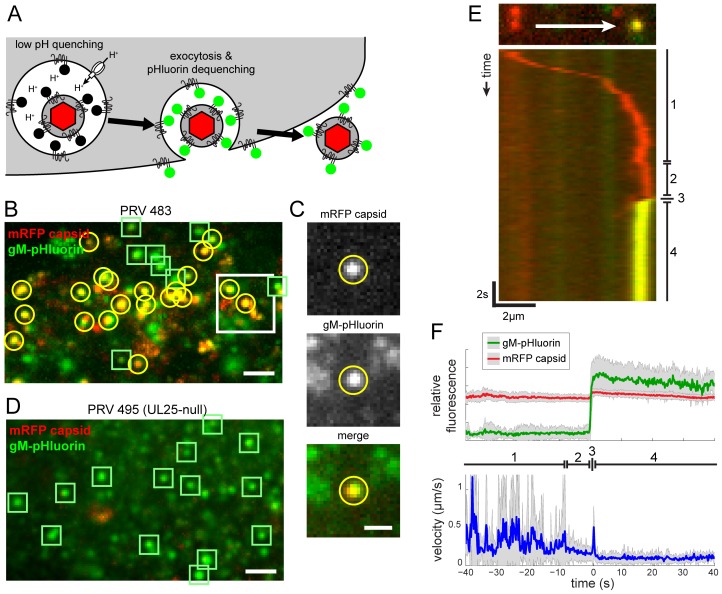
Live-cell fluorescence microscopy of particle transport and egress. (A) Schematic of virus particle transport and exocytosis assay. gM-pHluorin incorporated into virus particles or secretory vesicles is quenched in the acidic lumen of secretory vesicles (black circles), but the mRFP capsid tag is not (red hexagon). Upon exocytosis, pHluorin is exposed to neutral extracellular medium, and becomes fluorescent (green circles). (B) Virus particle exocytosis. PRV 483 infected cells were imaged at 4.5–5 hpi. Image is a maximum difference projection corresponding to Movie S1, depicting viral exocytosis events over a 13 min time course. Exocytosis of gM-pHluorin particles that do not contain capsids (green squares) and particles containing both gM-pHluorin and mRFP capsids (yellow circles) are indicated. Scale bar represents 2 µm. (C) Still images from Movie S1, depicting a single viral exocytosis event. Images correspond to the boxed area in panel B. Scale bar represents 1 µm. (D) Transcytosis of virus inoculum is almost never observed. Cells were infected with PRV 495 and imaged as in panel B. Image is a maximum difference projection corresponding to Movie S2, depicting exocytosis events over a 13 min time course. Exocytosis of gM-pHluorin particles that do not contain capsids are indicated by green squares. Particles containing mRFP capsids are almost never observed (not shown). Scale bar represents 2 µm. (E) Kymograph of a virus particle exocytosis event from Movie S1, depicting mRFP capsid (red) and gM-pHluorin (green) fluorescence over time. (F) Ensemble average of relative gM-pHluorin fluorescence (top, green line), mRFP capsid (top, red line), and instantaneous velocity (bottom, blue line) over 32 exocytosis events, in 10 cells, in 4 independent experiments. Shaded area represents standard deviation. (D and E) Virus particles exhibit stereotyped pattern of movement. (1) Fast directed transport. (2) Terminal pause. (3) Sharp jerk. (4) Mostly immobile.

Most intracellular membranes are acidified by the action of vacuolar ATPases, which pump protons from the cytoplasm into the lumen. Secretory vesicles are reported have a pH of 5.2–5.7 [16]. gM-pHluorin incorporated into virus particles or secretory vesicles is quenched at this acidic pH. However, when gM-pHluorin is exposed to the neutral extracellular medium upon exocytosis, particles exhibit a sharp, rapid increase in green fluorescence (Figure 2A).

We infected PK15 cells, a porcine kidney epithelial cell line, with PRV 483 and imaged by TIRF microscopy at 4.5–5 hours post-infection (hpi) to capture the earliest viral exocytosis events (Figure 2B and 2C), prior to the extensive changes in cell morphology and adherence that occur late in infection. Figure 2B corresponds to Movie S1, and represents a maximum difference projection over time to highlight areas where gM-pHluorin intensity increases rapidly. We identify three types of gM-pHluorin fluorescence patterns: (1) gM-pHluorin dequenches and then rapidly diffuses into the surrounding plasma membrane. This likely represents constitutive transport of gM to the plasma membrane. Importantly, we often observe such diffusion even during exocytosis of particles (Movie S1), indicating gM-pHluorin is incorporated into the secretory vesicle membrane as well as the particle envelope. (2) gM-pHluorin dequenches and remains punctate (Figure 2B and 2D, indicated by green squares). These events most likely represent exocytosis of membranous particles, such as L-particles, that do not contain capsids. At the beginning of the imaging time course, most gM-pHluorin puncta already present on the cell surface do not co-localize with capsids (Movie S1), suggesting that infected cells begin producing L-particles earlier than virions. (3) gM-pHluorin dequenches and is co-localized with mRFP capsids (Figure 2B, indicated by yellow circles). These events are the majority at this 4.5–5 hpi time point, and most likely represent exocytosis of virions.

Importantly, in nearly all cases, exocytosis events appear to release only single virions. Simultaneous exocytosis of multiple particles was observed only in a few instances out of the thousands of exocytosis events in more than 43 independent experiments.

### Exocytosis Events Represent Egress of Progeny, Not Transcytosis of Inoculum

Transcytosis is a process by which extracellular materials, including virus particles, can be taken up by endocytosis, retained in intracellular vesicles such as recycling endosomes, and subsequently released by exocytosis [17]. Accordingly, in our fluorescence microscopy method, viral exocytosis events could represent either egress of newly assembled progeny particles or transcytosis of the original inoculum. To determine the fraction of virus particle exocytosis events that are derived from transcytosed inoculum, we infected PK15 cells with PRV 495, which lacks the essential capsid protein UL25. In non-complementing PK15 cells, UL25-null viruses assemble capsids containing the mRFP capsid label (mRFP-VP26), but these defective capsids fail to exit the nucleus [18], [19]. As an internal positive control for viral protein expression, cell health, and function of the secretory pathway, we observed that PRV 495 produces L-particles that undergo exocytosis similarly to PRV 483 (Figure 2D, see also Movie S2). However, these particles almost never contained mRFP capsids (Figure 2D, indicated by green squares). Out of 165 exocytosis events in 20 cells, 3 independent experiments, and 2.6 hr total imaging time, we observed only one mRFP capsid undergoing exocytosis. Thus, under these infection and imaging conditions, greater than 99% of virus particle exocytosis events represent egress of newly synthesized progeny, and not transcytosis of inoculum.

### Particle Movement before, during, and after Exocytosis

We tracked virus particles and characterized their motion before, during, and after exocytosis. Figure 2E represents the movement and exocytosis of a single particle from Movie S1, and Figure 2F represents the ensemble average of many exocytosis events. We found that virus particles undergo a stereotyped pattern of movement, divided into four distinct phases (marked in Figure 2E and F). (1) Prior to exocytosis, particles exhibit fast, directional transport, with velocities up to 2 µm/s, directly to the site of exocytosis. This pattern of movement is consistent with the well-established role of microtubule motors in herpesvirus intracellular transport [20]–[22]. (2) Approximately 9 s before exocytosis, particles pause at the exocytosis site. This “terminal pause” or “docking” step is described in the cell biological literature, and likely represents the time required to form the molecular complexes that mediate vesicle fusion (e.g. [23]–[25]). (3) Within 200 ms of exocytosis, particles exhibit a sharp jerking movement (time resolution limited by the 200 ms exposure time). This motion appears as a small discontinuity on the kymograph of Figure 2E, and is represented by a spike in average velocity in Figure 2F. (4) After exocytosis, particles remain mostly immobile (Figure 2E and F).

#### Higher Time Resolution Imaging & MSD Analysis of Particle Movements

**Figure 3:**
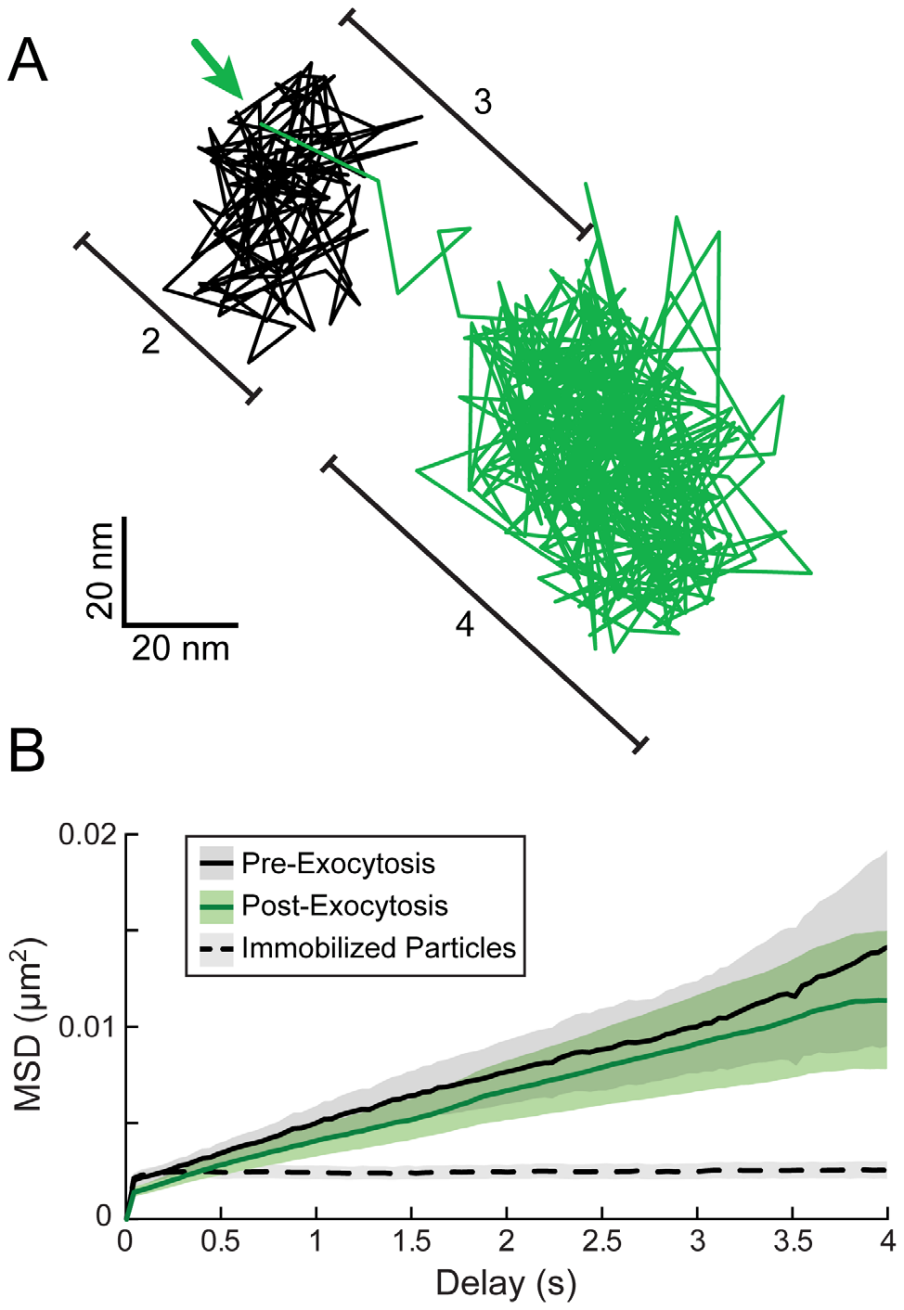
High time-resolution tracking and MSD analysis of particle movement. (A) PRV 483 infected cells were imaged at a rate of 25–50 frames/second, and particles were tracked before and after exocytosis. Graph shows one representative particle track, color-coded to indicate relative gM-pHluorin fluorescence. The location of gM-pHluorin dequenching is indicated (arrow). Bracketed regions correspond to pattern of movement indicated in Figure 2E and F: (2) Terminal pause. (3) Sharp jerk. (4) Mostly immobile. (B) Average MSD curves of particle tracks before and after exocytosis. Based on slope and MSD values, particles are confined an area approximately 400 nm in diameter before and after exocytosis (n = 43 exocytosis events, in 9 cells, in 3 independent experiments). Dotted MSD curve represents particles immobilized on glass (n = 249 particles). Shaded areas represent standard error of the mean.

We next performed higher time resolution imaging (25–50 frames/second), single-particle tracking, and mean squared displacement (MSD) analysis to better characterize particle motion in the few seconds before, during, and after exocytosis. As shown in a representative particle track (Figure 3A), particles appear to be confined both before and after exocytosis (color coded black and green, respectively). We segmented particle tracks at the moment of gM-pHluorin dequenching and performed MSD analysis to measure the spatial constraints of this confinement (Figure 3B). Based on the shape of MSD curves (not exponential), and the MSD values over 4 s (approximately 0.01 µm^2^), we calculate that particles are confined within an area approximately 400 nm in diameter both before and after exocytosis (Figure 3B). These observations suggest the following: Since virions are about 200 nm in diameter, these particles are confined inside secretory vesicles that are not much larger than the particle itself, consistent with observations by electron microscopy [26]. Importantly, those secretory vesicles are themselves docked at the plasma membrane in the seconds prior to exocytosis, unable to freely diffuse. After exocytosis, virus particles are retained at the cell surface, possibly by previously hypothesized mechanisms like the restriction factor tetherin [27], or viral glycoproteins binding to cell surface receptors like heparan sulfate proteoglycans [28].

Based on previous reports that myosin Va is involved in viral egress [29], the jerking movement we observed during exocytosis (Figure 2E and F) could represent the final push through cortical actin immediately prior to exocytosis. However, in higher time resolution analysis, we observed that this jerking movement consistently occurs after gM-pHluorin dequenching (Figure 3A), and therefore most likely represents post-fusion membrane relaxation. Particles moved an average of 116+/−75 nm (mean +/− standard deviation) from pre-fusion to post-fusion areas of confinement, which further suggests that the viral secretory vesicle is not much larger than the particle itself. While this observation does not preclude a role for myosin motors, we did not distinguish any other particle motion that could be attributed to transport through cortical actin.

### Rab6a, Rab8a, and Rab11a are Dynamically Associated with Virus Exocytosis

**Figure 4:**
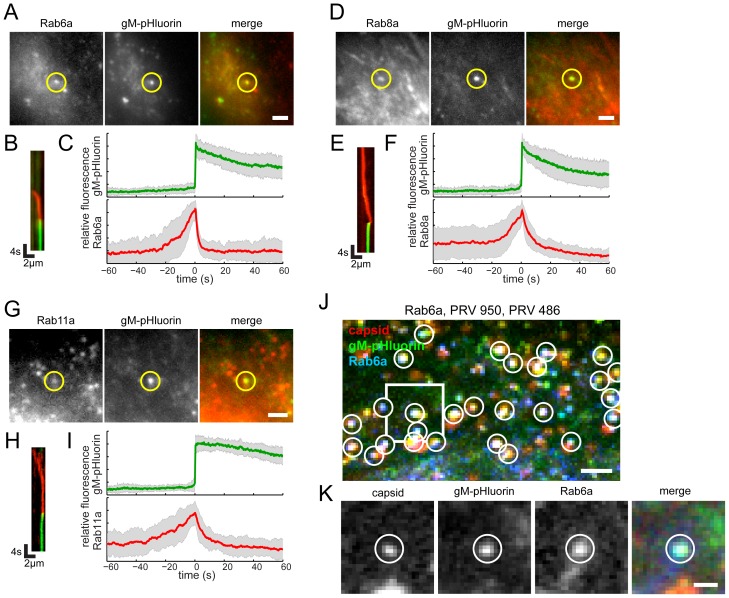
Rabs associated with virus particle exocytosis. Cells were transduced to express mCherry-tagged Rab proteins, infected with PRV 486 expressing gM-pHluorin, and imaged at 4.5–5 hr after PRV infection. (A,D,G) The indicated Rab proteins colocalize with gM-pHluorin particle at the moment of exocytosis (yellow circle). Images correspond to Movie S3. Scale bars represent 2 µm. (B,E,H) Kymographs of indicated Rab protein (red) and gM-pHluorin (green) fluorescence over time. (C,F,I) Ensemble averages of gM-pHluorin (top, green line) and indicated Rab protein (bottom, red line) relative fluorescence. Shaded area represents standard deviation. (A–C) mCherry-Rab6a. Data represent 37 exocytosis events in 4 independent experiments. (D–F) mCherry-Rab8a. Data represent 41 exocytosis events in 3 independent experiments. (G–I) mCherry-Rab11a. Data represent 34 exocytosis events in 3 independent experiments. (J–K) Rab6a is associated with exocytosis of assembled virions containing capsids. Cells were transduced to express mCherry-Rab6a, and co-infected with PRV 950 and PRV 486. (J) Image is a maximum difference projection corresponding to Movie S4, depicting virus particle exocytosis events over a 13.7 min time course. Exocytosis events associated with Rab6a (blue) containing gM-pHluorin (green) and capsids (red) are indicated (white circles). Scale bar represents 2 µm. (K) Still images from Movie S4, depicting a single viral exocytosis event. Images correspond to the boxed area in panel B. Scale bar represents 1 µm.

To characterize the viral secretory vesicle and identify some of the cellular proteins involved in viral egress, we transduced PK15 cells with non-replicating adenovirus vectors expressing red fluorescent protein-tagged Rab GTPases. Approximately 18 hr after transduction, we infected with PRV 486 and imaged 4.5–5 hr after PRV infection. We found that Rab proteins involved in the constitutive secretory pathway, Rab6a, Rab8a, and Rab11a, are present on the viral secretory vesicle immediately before exocytosis. After gM-pHluorin dequenching, the Rab proteins rapidly diffuse away from the site of exocytosis, consistent with the regulation of Rab activity and membrane binding by GTP hydrolysis (Figure 4B, E, & H; Movie S3). To generalize the relationship between fluorescent signals, we measured Rab fluorescence and gM-pHluorin fluorescence of many exocytosis events, aligned them to a common timescale based on moment of exocytosis, and calculated an ensemble average showing the relative fluorescence intensity over time (Figure 4C, F, & I). The increase in Rab fluorescence before exocytosis represents the gradual arrival of secretory vesicles to the site of exocytosis. After exocytosis, Rab fluorescence rapidly decays, representing the dissociation and diffusion of Rab proteins away from the site of exocytosis (Figure 4C, F, & I).

**Figure 5:**
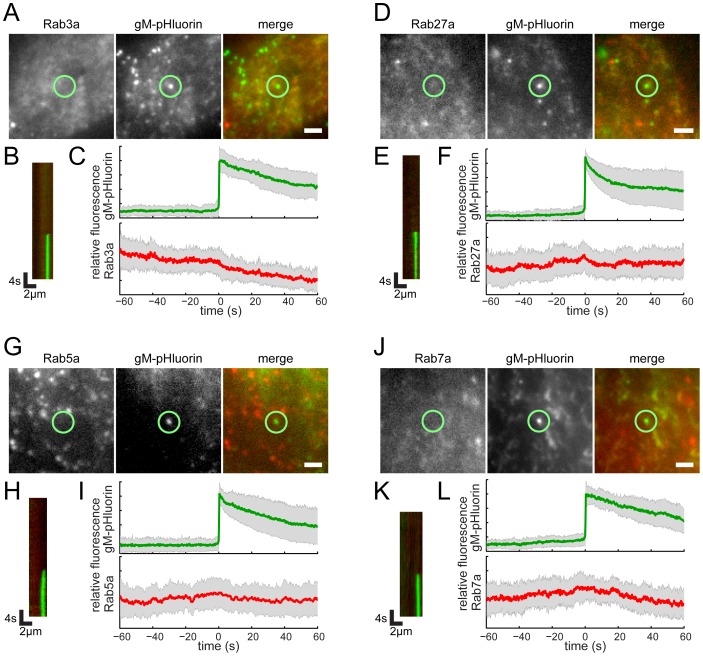
Rab proteins not associated with virus particle exocytosis. Cells were transduced to express mCherry-tagged Rab proteins, infected with PRV 486 expressing gM-pHluorin, and imaged at 4.5–5 hr after PRV infection. (A,D,G,J) The indicated Rab proteins are not present at gM-pHluorin exocytosis event (green circle). Images correspond to Movie S5. Scale bar represents 2 µm. (B,E,H,K) Kymographs of indicated Rab protein (red) and gM-pHluorin (green) fluorescence over time. (C,F,I,L) Ensemble averages of gM-pHluorin (top, green line) and indicated Rab protein (bottom, red line) relative fluorescence. Shaded area represents standard deviation. (A–C) mCherry-Rab3a. Data represent 38 exocytosis events in 2 independent experiments. (D–F) mCherry-Rab27a. Data represent 23 exocytosis events in 2 independent experiments. (G–I) mCherry-Rab5a. Data represent 37 exocytosis events in 2 independent experiments. (J–L) mCherry-Rab7a. Data represent 30 exocytosis events in 2 independent experiments.

Rab3a and Rab27a play important roles in regulated exocytosis in a variety of cell types; however, they do not appear to be associated with viral exocytosis in this cell type (Figure 5A–F, Movie S5). As a negative control, canonical early and late endosomal Rab proteins, Rab5a and Rab7a, also are not associated with viral exocytosis (Figure 5G–L, Movie S5).

A recent study from the Elliott laboratory reported that Rab6 is involved in viral membrane protein trafficking prior to HSV-1 assembly [6]. Therefore, to determine whether the Rab6a-associated exocytosis events we observed in Figure 4A represent egress of assembled virions or intracellular trafficking of gM-pHluorin alone, we performed three-color experiments to simultaneously image Rab6a, gM-pHluorin, and capsids. We transduced PK15 cells with an adenovirus vector expressing red fluorescent Rab6a, and subsequently co-infected these cells with PRV 486, expressing gM-pHluorin, and PRV 950, expressing a cyan fluorescent VP26 capsid protein. Under these conditions, nearly all gM-pHluorin exocytosis events were associated with Rab6a, and co-localized with fluorescent capsids (Figure 4J and K), indicating that Rab6a is involved in exocytosis of assembled virus particles that do contain capsids.

### Viral Exocytosis Sites Are Not Depleted of Cytoskeleton Components

**Figure 6:**
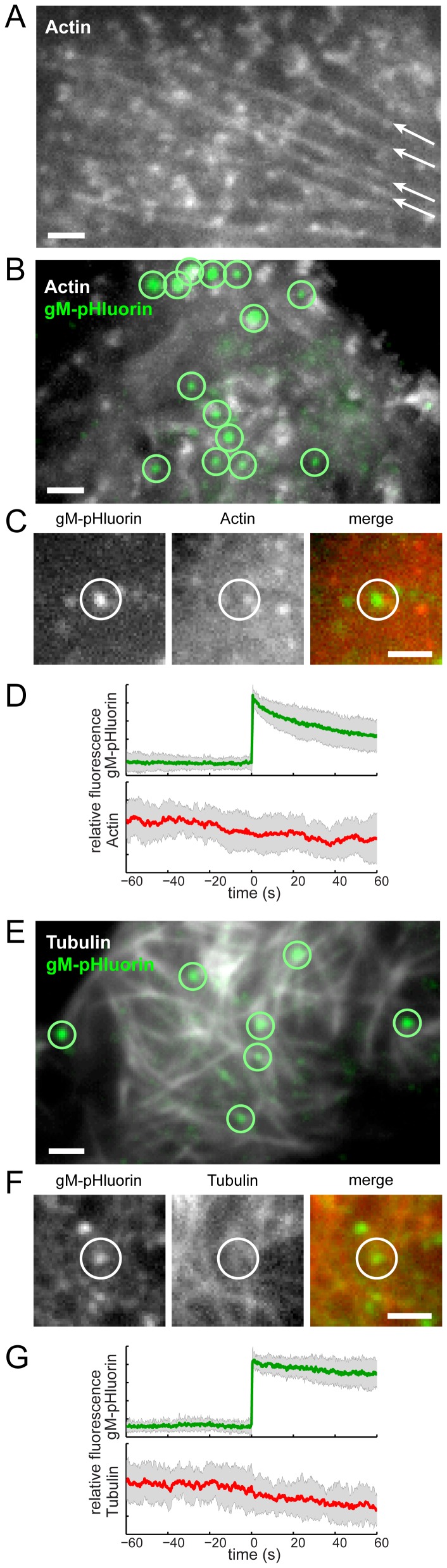
Viral exocytosis does not occur at specialized sites depleted in cytoskeleton proteins. (A) Cells were transduced to express mCherry-actin. Long actin stress fibers are indicated (arrows). (B) Cells were transduced to express mCherry-actin, infected with PRV 486 expressing gM-pHluorin, and imaged at 4.5–5 hr after PRV infection. The image is a maximum difference projection depicting virus particle exocytosis events (green circles) over a 10.5 min time course. (C) Still images depicting a single exocytosis event in panel B. (D) Ensemble averages of gM-pHluorin (top, green line) and mCherry-actin (bottom, red line) relative fluorescence. Shaded area represents standard deviation. Data represent 34 exocytosis events in 3 independent experiments. (E) Cells were transduced to express mCherry-tubulin and infected with PRV as above. The image is a maximum difference projection depicting virus particle exocytosis events (green circles) over a 9 min time course. (F) Still images depicting a single exocytosis event in panel E. (G) Ensemble averages of gM-pHluorin (top, green line) and mCherry-tubulin (bottom, red line) relative fluorescence. Shaded area represents standard deviation. Data represent 24 exocytosis events in 3 independent experiments. Scale bars represent 2 µm in all images.

A previous study reported accumulation of HSV-1 particles at specialized plasma membrane invaginations depleted of cytoskeleton components, including actin filaments and microtubules [10]. To investigate whether specialized cytoskeleton depleted egress sites are apparent in our experimental system, we transduced PK15 cells with adenovirus vectors expressing red fluorescent protein-tagged actin or tubulin, infected with PRV 486, and imaged at 4.5–5 hr after PRV infection. In agreement with many previous reports (reviewed in [30], [31]), we observe a global dysregulation of the actin cytoskeleton. Long actin stress fibers apparent in uninfected cells (Figure 6A, arrows) are not visible in cells infected with PRV, but smaller punctate actin structures remain (Figure 6B). However, this global effect aside, we find no relationship between gM-pHluorin exocytosis events and local actin fluorescence intensity (Figure 6B, and C), nor changes in actin fluorescence during exocytosis (Figure 6D).Similarly, we find no evidence for local depletion of microtubules (Figure 6E and F), and no changes in microtubule fluorescence during exocytosis (Figure 6G).

### Exocytosis of Virus Particles Occurs Most Frequently Near LL5β Patches

It was reported previously that exocytosis occurs most frequently near plasma membrane patches of LL5β, or its binding partner ELKS/ERC1, members of a molecular complex that anchors microtubules to the plasma membrane. Ohara-Imaizumi et al observed insulin exocytosis events near ELKS/ERC1 patches in a pancreatic β cell line [32], and the Akhmanova laboratory observed exocytosis of Rab6a-positive vesicles near LL5β patches in HeLa cells [24]. In both of these studies, ∼80% of exocytosis events occurred near LL5β or ELKS/ERC1 patches on the plasma membrane.

**Figure 7:**
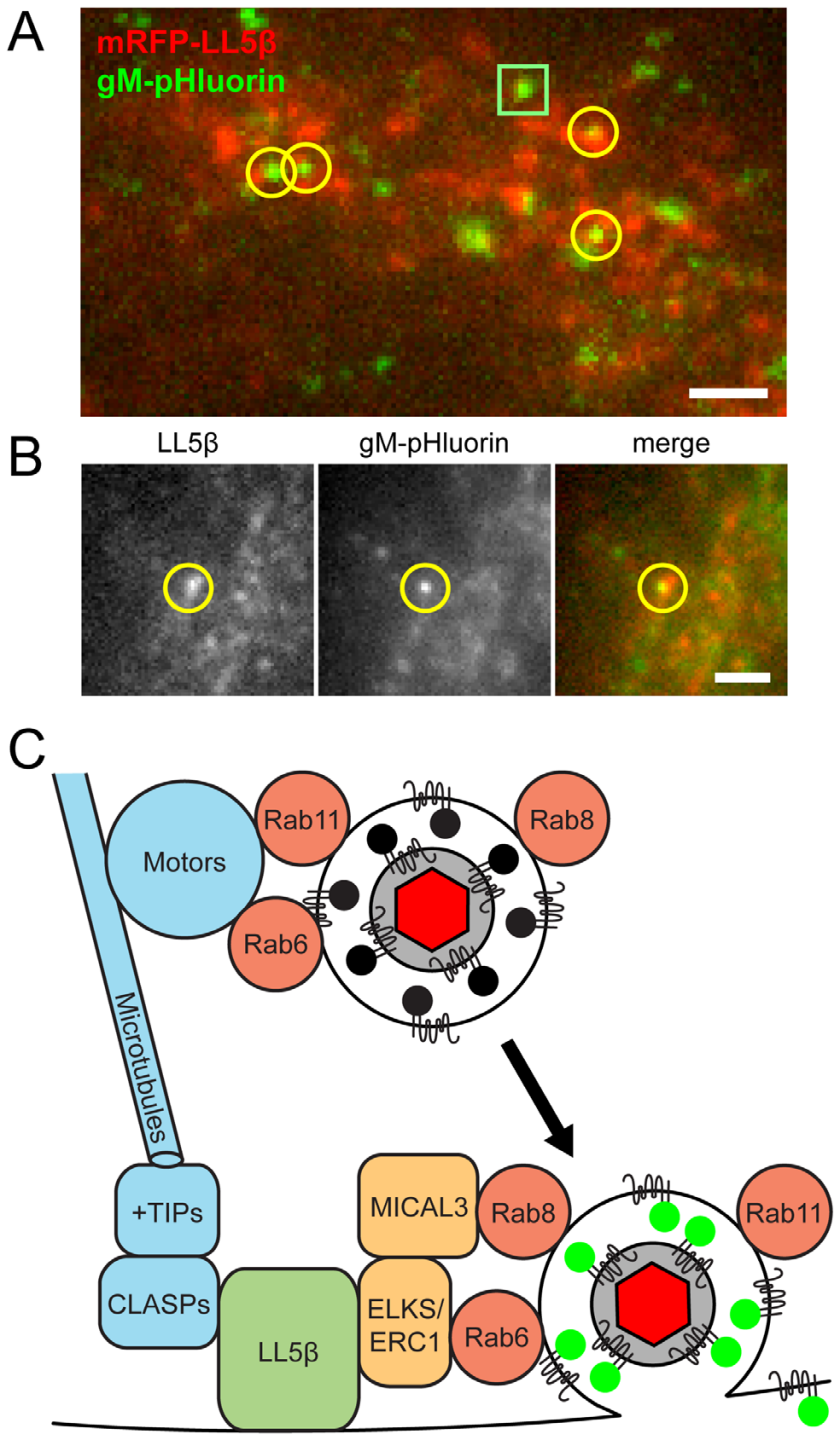
Viral exocytosis occurs most frequently near patches of LL5β. (A–B) Cells were transduced to express mRFP-LL5β, infected with PRV 486 expressing gM-pHluorin, and imaged at 4.5–5 hr after PRV infection. Data represent 150 exocytosis events in 9 independent experiments. Scale bars represent 2 µm in all images. (A) Image is a maximum difference projection depicting exocytosis events over a 10 min time course. Particle exocytosis events are classified according to their proximity to mRFP-LL5β patches (yellow circles), or lack thereof (green squares). (B) Still images of a single exocytosis event, corresponding to Movie S6. (C) Schematic of molecular and cellular mechanisms that coordinate viral transport and exocytosis. Please refer to the discussion section for references supporting the depicted molecular links.

To determine whether PRV particle exocytosis also occurs near LL5β patches, we transduced PK15 cells with adenovirus vectors expressing red fluorescent protein-tagged LL5β, infected with PRV 486, and imaged at 4.5–5 hr after PRV infection, as described above. We identified particle exocytosis events by gM-pHluorin dequenching, and classified exocytosis events according to their full or partial overlap with LL5β puncta (Figure 7A and B, yellow circles). Out of 150 exocytosis events in 34 cells, 9 independent experiments, and 2.25 hr total imaging time, we found 83% of exocytosis events occurred at or immediately adjacent to LL5β patches on the plasma membrane, consistent with the cell biological literature described above [24], [32].

## Discussion

Egress of alpha herpesvirus particles from infected cells is a complex, multistep process. In this study, we have established a new vantage point from which to describe the dynamics of this process, characterize the exiting particle, and identify molecular and cellular mechanisms involved in virus egress. Based on our data, individual virus particles travel to the plasma membrane inside small, acidified secretory vesicles decorated with Rab6a, Rab8a, and Rab11a. These vesicles undergo fast, directional transport on microtubules directly to the site of exocytosis, most frequently plasma membrane LL5β complexes. Upon arrival, vesicles are tightly docked at the site of exocytosis for several seconds. Membrane fusion occurs, displacing the virion a small distance across the plasma membrane. In nearly all cases, single virus particles are released, rather than multiple particles per exocytosis event. After exocytosis, particles remain tightly attached to the outer cell surface.

### Are There Specialized Virus-Induced Egress Sites?

Our work contradicts an earlier study that used TIRF microscopy to investigate the distribution of HSV-1 particles on the surface of infected cells [10]. The Brown laboratory imaged fixed cells primarily at 12 hpi, and did not directly observe individual virus exocytosis events. Rather, they observed plasma membrane invaginations containing many virus particles, which they inferred to be sites of virus egress. It is possible that these structures represent sites of preferential particle accumulation late in infection, and may form well after the actual particle exocytosis events. The authors reported that these structures are depleted of cytoskeleton elements, including actin and microtubules. However, the use of TIRF microscopy comes with an important caveat: the TIRF excitation field selectively excites fluorescent molecules within a few hundred nanometers of the coverslip, so only the adherent “footprint” of the cell can be observed. Since the plasma membrane invaginations observed by the Brown lab are up to a micrometer deep, it is likely that cytoskeleton proteins were outside of the TIRF excitation field, rather than biologically depleted. We observe virus particle exocytosis frequently occurs near LL5β complexes, as has also been reported for cell biological cargoes. Thus, our data favor a model in which virus egress sites are specified primarily by cellular mechanisms, rather than induced by viral processes.

### Can We Infer the Origin and Trafficking Route of Virus Particle Secretory Vesicles?

Rab6 is primarily associated with the Golgi and is present on post-Golgi secretory vesicles [24], [33], whereas Rab11 is typically, but not exclusively, associated with recycling endosomes [34]. Recent studies from the Elliott laboratory showed that siRNA knockdown of either Rab6 or Rab11 caused defects in secondary envelopment, resulting in a significant decrease in infectious titer (>100-fold or>10-fold reduction, respectively) [5], [6]. In their proposed model, viral glycoproteins traffic via the plasma membrane to recycling endosomes, which form the membrane of secondary envelopment. Accordingly, their model suggests two separate exocytosis routes in viral assembly and egress: viral glycoproteins take a Rab6-dependent route from the trans-Golgi to the plasma membrane, and assembled virions take a Rab11-dependent route from recycling endosomes to the plasma membrane. In contrast, we do not observe any such distinction: both Rab6 and Rab11 are associated with exocytosis of particles, and we confirmed that Rab6 is associated with exocytosis of assembled virions containing capsids (Figure 4).

While Rab6 and Rab11 are associated with post-Golgi transport and recycling endosomes, respectively, Rab8 functions in both. Rab8 is associated with Golgi-derived secretory vesicles [35], [36] in combination with Rab6 [23], but it is also associated with recycling endosomes in combination with Rab11 [34]. Knockdown of Rab8 had a small effect, not statistically significant [6], thus it may be a minor player in these processes. Yet, in light of these overlapping functions, whether virus secretory vesicles are derived directly from trans-Golgi or recycling endosomes cannot be ascertained based on Rab proteins alone.

### Model of Cellular Factors That Link Virus Particle Transport and Exocytosis

Combining recent reports in the literature with our spatial and dynamic data on viral egress, we propose an integrated model of the molecular interactions that link the final transport and exocytosis steps during egress (Figure 7B):

First, Rab proteins may promote intracellular transport by recruiting microtubule motors. Rab6a and Rab11a have been reported to interact with kinesins (kinesin-1 and rabkinesin-6) and dynein [7], [24]. Although viral membrane and tegument proteins also interact with microtubule motors [21], [37], siRNA knockdown of several individual motor proteins has little impact on production of infectious alpha herpesvirus [6]. It may be that these Rab proteins contribute to functional redundancy in motor recruitment, ensuring efficient intracellular transport of virus particles during egress (Figure 7B).

Second, LL5β complexes anchor stabilized microtubules to the plasma membrane. Naghavi et al. recently reported that HSV-1 infection induces the formation of stabilized microtubules that run from the trans-Golgi to the plasma membrane [38]. These microtubules are regulated by microtubule plus-end tracking proteins (+TIPs), and are anchored to the plasma membrane by cytoplasmic linker associated proteins (CLASPs) through a complex with LL5β [39]. Naghavi et al. showed that siRNA knockdown of CLASPs impairs efficient alpha herpesvirus production and cell-cell spread [38]. Thus, stabilized microtubules, anchored at the plasma membrane by LL5β, may provide an efficient pathway linking the site of secondary envelopment to the site of egress (Figure 7B).

Finally, after arrival to the plasma membrane, virus particle exocytosis occurs most frequently near patches of LL5β. The Akhmanova laboratory showed that this preferential exocytosis depends on molecular interactions between Rab6a and Rab8a with LL5β complexes, via ELKS/ERC1 and MICAL3, respectively [23], [24]. Importantly, Johns et al. showed that siRNA knockdown of ELKS/ERC1 impairs HSV-1 production 5–10 fold [6]. Thus, the LL5β complex plays a central role, linking intracellular transport to exocytosis during the final steps in alpha herpesvirus egress (Figure 7B).

### Future Directions

In this study, we investigated alpha herpesvirus egress in non-polarized epithelial cells that perform constitutive exocytosis. Initial alpha herpesvirus infections typically begin at epithelial cell surfaces, but infection subsequently spreads to polarized cells with highly specialized regulated secretory pathways. VZV infects T cells [40], which perform regulated exocytosis of cytokines and cytotoxic granules at the immunological synapse [41]. All alpha herpesviruses infect sensory and autonomic neurons [42]. Neurons have highly specialized secretory pathways, and exocytosis of neurotransmitters and neuropeptides is highly regulated at neuronal synapses. In addition to their role in constitutive exocytosis, LL5β complexes mediate regulated exocytosis at presynaptic active zones in neurons [43]. Rab3 and Rab27 are associated with regulated exocytosis in many cell types, including neurons and T cells [44], and are reported to be involved in HSV-1 transport and replication in neurons and oligodendrocytes, respectively [45], [46]. Despite these clues, the relationship between constitutive and regulated secretory mechanisms and alpha herpesvirus assembly and egress in these cell types is not known, and is an important topic for future studies.

## Materials and Methods

### Cell Culture, Transfection, and Virus Propagation

PK15 cells and 293A cells (Invitrogen) were cultured in Dulbecco's modified Eagle's medium (DMEM) supplemented with 10% fetal bovine serum (FBS), and penicillin/streptomycin. To construct recombinant viruses and vectors, cells were co-transfected with plasmid DNA and/or virus nucleocapsid DNA using Lipofectamine 2000 (Invitrogen). PRV recombinants were propagated on PK15 cells in DMEM with 2% FBS, penicillin/streptomycin, and 20 mM HEPES buffer. Infectious virus concentration was measured by serial dilution plaque assay. Adenovirus vectors were propagated on complementing 293A cells in DMEM with 2% FBS. Vector stocks were prepared by discarding supernatants and resuspending cells and debris in DMEM with 20 mM HEPES without serum, and freezing three times in liquid nitrogen. The transduction efficiency of adenovirus vectors was determined by quantitating fluorescent protein expression in non-complementing PK15 cells.

### Single-Step Replication Assay

PK15 cells were seeded into each well of 6-well plates and grown for 24 h. PRV recombinants were inoculated in triplicate at a multiplicity of infection (MOI) of 5 plaque-forming units per cell, incubated at 37°C for 1 hr, and thoroughly washed three times with phosphate-buffered saline (PBS). Cells and supernatants were harvested at 4, 6, 8, 12, and 24 hpi, and titered in duplicate by serial dilution plaque assay.

### Construction of PRV Recombinants

All PRV recombinants are derivatives of PRV Becker. PRV 180, expressing mRFP-VP26 small capsid protein, was previously described [12]. PRV 950, expressing mTurquoise2-VP26, was constructed as follows: Plasmid pGS397 [12] contains an EGFP fusion to *UL35* (VP26). We replaced EGFP with mTurquoise2 fluorescent protein, derived from pmTurquoise2-Tubulin [47], obtained from D. Gadella via Addgene (#36202). The resulting plasmid, pmTurquoise2-vp26, was co-transfected with PRV 180 nucleocapsid DNA, and plaques were screened for loss of mRFP and gain of mTurquoise2 expression.

PRV 130 and PRV 134 were constructed by recombination in E. coli using pBecker3, a bacterial artificial chromosome containing the PRV Becker genome [48]. PRV 130, a gM-null strain, contains nonsense mutations at codons 8 and 53 of the *UL10* (gM) gene. PRV 134 expresses gM with a C-terminal EGFP fusion (gM-EGFP). PRV 137, expressing both gM-EGFP and mRFP-VP26, was created by co-infecting PRV 134 and PRV 180, and screening plaques for mRFP and EGFP co-expression (L. Pomeranz, S. Bratman, and L. Enquist, unpublished data).

PRV-GS2168, a *UL25*-null mutant expressing mRFP-VP26, and a complementing cell line expressing PRV UL25 protein, were a kind gift from G. Smith, Northwestern University [49].

The plasmid pUC57-EC1-gM-pHluorin was used to construct all recombinant viruses expressing gM-pHluorin. pUC57-EC1-gM-pHluorin was synthesized (GenScript) to contain codon-optimized super-ecliptic pHluorin fluorescent protein sequence, flanked on each side by 250 bp of the *UL10* (gM) gene, designed to insert pHluorin into the first extracellular loop of gM. The fusion junction is as follows (gM sequence is in bold type, peptide linkers are italicized, and pHluorin sequence are in plain type): … **LFLETPTVTS** *GGGTG*SKGEEL…MDELYK*GGSGG* **VTSFFGFTAT** …

We constructed PRV 483 by co-transfecting pUC57-EC1-gM-pHluorin with PRV 180, and screened plaques for gain of pHluorin expression. We constructed PRV 486 by co-infecting PRV 483 with parental PRV Becker, and screened plaques for loss of mRFP fluorescence. We constructed PRV 495 by co-transfecting pUC57-EC1-gM-pHluorin with PRV-GS2168 DNA, and screened recombinants that gained pHluorin expression.

### Construction of Adenovirus Vectors

All vectors are based on human adenovirus type 5 (Ad5) containing deletions in the E1 and E3 regions. The vector expressing mCherry-tagged α-tubulin, pAdEasy-mCherry-Tubulin [50], was obtained from Addgene (#26767).

All other vectors were constructed by PCR amplification followed by Gateway recombination into pAd/CMV/V5-DEST (Invitrogen). We constructed an mCherry-tagged β-actin construct based on plasmid pAcGFP1-actin (Clontech). The coding sequences of Rab3a and Rab27a were kind gifts from M. Fukuda [51]. Rab5a and Rab11a were obtained from A. Ono, University of Michigan. Rab7a was obtained from R. Pagano [52] via Addgene (#12605). Rab8a was obtained from M. Nachury [53], via Addgene (#24898). In each case, mCherry was fused to the Rab GTPase as follows (the C-terminus of mCherry and the first methionine of the Rab are represented in bold type, and a linker peptide is in plain type): … **MDELYK**GTTLYTKVGS**M** … A plasmid encoding mCherry-Rab6a was synthesized (GenScript) based on UniProtKB accession number P20340. mCherry was fused to Rab6a as follows (the C-terminus of mCherry and the first methionine of Rab6a are represented in bold type, and a linker peptide is in plain type): … **MDELYK**SGGSGGTGGS**M** … A plasmid encoding mRFP-tagged LL5β was a kind gift from A. Akhmanova [39].

### Western Blot

PK15 cells were infected with PRV recombinants at an MOI of 5. At 12 hpi, cell lysates were harvested and analysed by SDS-PAGE and Western blot, as previously described [54]. Parallel blots were probed with Ab183 polyclonal antiserum against PRV gM [13], and a monoclonal anti-GFP antibody that recognizes both EGFP and pHluorin variants (Roche). Blots were subsequently probed with a horseradish peroxidase-conjugated secondary antibody and visualized by chemiluminescence, as previously described [54].

### Membrane Topology and pH Dependence

PK15 cells were seeded into 6-well cell culture dishes and grown until confluent. Cells were then inoculated with PRV 134, PRV 137, PRV 483, or PRV 486 at an MOI of 5. At 12 hpi, supernatants were collected and immediately spotted onto coverslips mounted in 35 mm cell culture dishes (Mat-Tek). Individual particles were imaged using a previously described epifluorescence microscope [55] with a Plan Apo 60×/1.40 NA oil immersion objective (Nikon). Fluorescence excitation and emission bands are as follows: EGFP and pHluorin, 490/20 nm excitation, 526/36 nm emission; mRFP, 555/25 nm excitation, 632/60 nm emission; Alexa647, 645/30 nm excitation, 705/72 nm emission. For membrane topology experiments, coverslips were incubated for 15 min in a 1∶100 dilution of a polyclonal anti-GFP antibody that recognizes both EGFP and pHluorin variants (Clontech), followed by 15 min in a 1∶200 dilution of anti-rabbit AlexaFluor 647 secondary antibody (Invitrogen). For pH dependence experiments, particles were imaged in DMEM (pH∼7.4), followed by sequential washes of MES-buffered saline (154 mM NaCl and 0.1M morpholinoethanesulfonic acid in deionized H_2_O, pH 6.0), and PBS (pH 7.1). Particle intensities were measured using the Analyze Particles function in Fiji/ImageJ, version 1.48 [56].

### TIRF Microscopy

The two-color, live-cell TIRF movies were all acquired using a custom-built microscope in the Princeton University Lewis-Sigler Imaging Core Facility, consisting of the following components: 488 nm (Coherent) and 561 nm (CrystaLaser) excitation lasers, an acousto-optical tunable filter (AA Optoelectronic), a Plan Apo 60×/1.49 NA oil immersion objective (Olympus), a 37°C heated stage and coverslip holder, a multiband filter set (Semrock, LF488/561-A-000), an Andor iXon EMCCD camera, and custom control software written in Matlab (Mathworks). Fluorescence emission bands are as follows: pHluorin, 523/40 nm emission; mRFP, 610/52 nm emission.

Three-color, live-cell TIRF movies were acquired on a Nikon Ti-E microscope in the Princeton University Molecular Biology Confocal Microscopy Facility. This microscope is equipped with 405 nm, 488 nm, and 561 nm excitation lasers (Agilent), an Apo TIRF 100×/1.49 NA oil immersion objective (Nikon), an Andor iXon Ultra EMCCD camera, a 37°C heated stage, and Nikon NIS Elements software. Fluorescence emission bands are as follows: mTurquoise2, ∼450/60 nm; pHluorin, ∼525/50 nm emission; mRFP, ∼605/50 nm emission.

### MSD Analysis

Single particle tracking was performed in Fiji/ImageJ using MTrackJ [57] plugins. Particle location and track data was imported into Matlab and mean squared displacements were calculated using MSDAnalyzer [58]. Particle corral size was calculated by 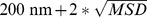, where 200 nm is the approximate virion diameter, and *MSD* represents the average mean squared displacement over 4 seconds.

### Image Processing

Images were prepared for publication using the following functions and plugins in Fiji/ImageJ: adjust brightness and contrast, Kalman filter (to reduce noise in timecourse microscopy images), Z project (to make maximum intensity projections), dynamic reslice (to make kymographs), and save as AVI (to export movies). We calculated maximum difference projections in Fiji/ImageJ as follows: Image values at time n were subtracted from values at time n+5 to identify pixels where fluorescence intensity increases. The resulting image stacks were then processed by maximum intensity projection to highlight areas where fluorescence intensity increases the most. In these image sets, maximum difference projection identifies gM-pHluorin exocytosis events where fluorescence intensity rapidly increases.

## Supporting Information

Movie S1 **Exocytosis of PRV particles.** This movie corresponds to Figure 2B.
(AVI)

Movie S2 **PRV exocytosis events are progeny, not transcytosis of inoculum.** This movie corresponds to Figure 2D.
(AVI)

Movie S3 **Virus exocytosis is associated with Rab6a, Rab8a, and Rab11a.** This movie corresponds to Figure 4A, D, & G.
(AVI)

Movie S4 **Rab6a is associated with exocytosis of assembled virions containing capsids.** This movie corresponds to Figure 4J.
(AVI)

Movie S5 **Virus exocytosis is not associated with Rab3a, Rab27a, Rab5a, or Rab7a.** This movie corresponds to Figure 5A, D, G, & J.
(AVI)

Movie S6 **Virus exocytosis most frequently occurs near plasma membrane patches of LL5β.** This movie corresponds to Figure 7B.
(AVI)
